# Clinical and demographic profile of catatonic patients who received electroconvulsive therapy in a South African setting

**DOI:** 10.4102/sajpsychiatry.v24i0.1100

**Published:** 2018-08-30

**Authors:** Kavendren Odayar, Ingrid Eloff, Willem Esterhuysen

**Affiliations:** 1 Department of Psychiatry, Walter Sisulu University, South Africa

## Abstract

**Background:**

Catatonia is a psychomotor dysregulation syndrome seen in several illnesses. Uncertainties exist regarding its prevalence and causes. While some research shows a strong association with mood disorders, other data show catatonia to be strongly associated with schizophrenia. Data from low- and middle-income countries are required.

**Aim:**

To determine the clinical and demographic profile of patients with catatonia that received electroconvulsive therapy (ECT) between 01 January 2012 and 31 December 2014.

**Setting:**

The study was conducted at Elizabeth Donkin Psychiatric Hospital in Port Elizabeth, Eastern Cape. The hospital has mostly patients admitted under the *Mental Health Care Act* 17 of 2002 as Involuntary Mental Health Care Users.

**Method:**

A retrospective chart review was conducted. Using the hospital ECT database, all files of patients who received ECT for catatonia were identified. Demographics, psychiatric and medical diagnoses, signs of catatonia and other data were abstracted from these files.

**Results:**

Forty-two patients received ECT for catatonia, of whom 34 (80.95%) were diagnosed with a psychotic illness. Schizophrenia was the most common diagnosis (*n* = 19; 45.24%), followed by psychotic disorder owing to a general medical condition (*n* = 8; 19.05). Human immunodeficiency deficiency virus was the cause in 75.00% of the patients whose medical conditions caused catatonia. Seven (16.67%) patients had mood disorders, with bipolar I disorder accounting for 6 (14.29%) of these.

**Conclusion:**

Psychotic disorders were more frequent than mood disorders in the sample. Schizophrenia was the most common diagnosis, followed by psychotic disorder owing to a general medical condition.

## Introduction

Catatonia is a psychomotor dysregulation syndrome seen in several illnesses.^1^ The prevalence of catatonia is unclear,^2^ and it is thought to be under-recognised and under-diagnosed.^3^ According to the Diagnostic and Statistical Manual of Mental Disorders, Fifth Edition (DSM 5), catatonia is typically diagnosed in patients admitted to hospital.^4^ The results of 10 international prospective studies indicate that catatonia is seen in 10% of hospital admissions.^5^

The 3 principal subtypes of catatonia are retarded catatonia, excited catatonia and malignant catatonia.^6^ Patients with retarded catatonia show a marked reduction in movement despite preserved physical ability to move^2^ and impairments may be seen with the initiation and cessation of movement.^7^ Patients with excited catatonia show agitation,^2^ frequently with disturbed purposeless movement.^7^ Retarded and excited catatonia may co-exist, with patients fluctuating between the 2 extremes.^8^ Malignant catatonia is a life-threatening condition, characterised by fever, autonomic instability, delirium and rigidity.^2^ Most patients present with the retarded subtype.^9^

Clinical features which may be present in patients with catatonia include those listed in Box 1.

Box 1 Clinical features of catatonia.

1. Excitement
2. Immobility or stupor
3. Mutism
4. Staring
5. Posturing
6. Catalepsy
7. Grimacing
8. Stereotypies
9. Mannerisms
10. Verbigeration
11. Rigidity
12. Negativism
13. Waxy flexibility
14. Echolalia
15. Echopraxia
16. Withdrawal
17. Impulsivity
18. Automatic obedience
19. Perseveration
20. Combativeness
21. Autonomic changes

*Source:* Daniels^1^

Catatonia may be associated with neurodevelopmental disorders, psychotic illnesses, bipolar disorders, depressive disorders and medical illnesses.^4^ Research findings suggest that underlying causes may differ in different populations. Some data suggest a strong association with mood disorders,^10^ while other studies indicate that catatonia is associated with schizophrenia and other psychotic disorders.^11^ There is a need for research related to catatonia and its treatment in low- and middle-income countries (LMICs).^12^ Determining the underlying cause is essential, as it requires specific treatment.^5^

Management of catatonia itself includes supportive care, pharmacological treatments and electroconvulsive therapy (ECT).^8^ There is evidence to indicate that catatonia may be treated with benzodiazepines alone, ECT alone or ECT in combination with benzodiazepines.^10^ Supportive care aims to reduce the risk of morbidity and mortality caused by immobility and poor nutrition.^1^ The benzodiazepine most commonly used to treat catatonia is lorazepam.^5^ Whether certain benzodiazepines may work better than others has not been carefully investigated.^9^ Electroconvulsive therapy has been found to be the most effective treatment for catatonia independent of the underlying cause.^13^ Bitemporal placement of electrodes is favourable and a cycle of at least 6 sessions is recommended.^5^

Investigation of the distribution and features of catatonia locally will aid in understanding the causes and associations of the syndrome in this setting. This is important, as early identification will allow for timely initiation of treatment for both the catatonia and the underlying cause.^5,14^ We conducted a retrospective review of patients admitted to Elizabeth Donkin Hospital in Port Elizabeth, South Africa, in order to determine the underlying diagnoses in hospitalised patients receiving ECT for catatonia.

## Aim

To determine the clinical and demographic profile of patients with catatonia who received ECT at Elizabeth Donkin Hospital, Port Elizabeth, between 01 January 2012 and 31 December 2014.

## Research methods and design

### Study design

This study was a retrospective descriptive chart review. It was a cross-sectional survey and was non-experimental in nature.

### Study setting

The study was conducted in Port Elizabeth at Elizabeth Donkin Hospital, which is a designated psychiatric hospital providing care, treatment and rehabilitation services for patients with mental illness.^15^ The majority of patients at the hospital are admitted under the *Mental Health Care Act* 17 of 2002 as Involuntary Mental Health Care Users.^15^ The predominant illnesses seen are schizophrenia and related disorders, bipolar and related disorders, as well as substance use disorders with associated neuropsychiatric manifestations. Neuropsychiatric manifestations associated with medical conditions are also frequently seen.

Electroconvulsive therapy services at the hospital are provided when clinically indicated. All the relevant Mental Health Care Act forms are completed when the ECT is administered. Patients with possible catatonia are presented by medical officers or registrars at a consultant-driven multidisciplinary team ward round. Those diagnosed with catatonia are administered a trial of lorazepam; ECT is initiated if there is a response to lorazepam. The dose of lorazepam is reduced while the patient receives ECT, and definitive management is initiated before completion of ECT. Electroconvulsive therapy is stopped once the signs of catatonia have resolved, and this is based on clinical evaluation. A consultant report and hospital ECT document are completed for all patients receiving ECT; this includes information on clinical findings, whether or not the patient meets DSM criteria for catatonia, diagnostic information, a recommended management plan, the indication for ECT, medical comorbidities and results of special investigations. Both the consultant report and the ECT document are filed in the relevant patient folders.

### Study population

Names and folder numbers of patients who receive ECT at Elizabeth Donkin Hospital are recorded in an electronic database which was used to identify all patients who received ECT during the study period. The hospital folders of these patients were retrieved and reviewed. All patients who were administered ECT for catatonia were included in the study.

### Data abstraction

Information from the consultant psychiatrist’s reports and the medical officer’s clinical notes was recorded on a data collection sheet. Each data collection sheet was numbered.

Demographic variables, including age, sex, and race, were abstracted. The clinical signs of catatonia were categorised into the Diagnostic and Statistical Manual of Mental Disorders, Fourth Edition, Text Revision (DSM-IV-TR) categories:^16^

- Motoric immobility, including catalepsy or stupor.
- Excessive motor activity that is purposeless and not influenced by external stimuli.
- Extreme negativism or mutism.
- Peculiarity of voluntary movements such as posturing, stereotyped movements, mannerisms or grimacing.
- Echolalia or echopraxia.

Signs of catatonia which did not fall into one of these categories were recorded separately. The final DSM-IV-TR psychiatric diagnosis, medical morbidities, information regarding illicit substance use and the number of ECTs received were also abstracted.

### Analysis of data

Analysis was carried out using SAS Version 9.2. Descriptive statistics, namely, frequencies and percentages, were calculated for categorical data; means or medians were calculated for numerical data. Analysis focused on the description of demographic and clinical profiles as well as the profile of catatonic signs. Comparative analyses focused on comparing the prevalence of different catatonic signs with the underlying diagnoses, and on comparing demographic characteristics, diagnostic factors and substance use with the number of ECTs received and the length of hospital stay. Analytical statistics, namely, the chi-square test (or Fisher’s exact test) for nominal data and the Kruskal-Wallis test for ordinal data, was used. A significance level of 0.05 was used.

## Ethical consideration

The data were recorded on a data collection sheet by the researcher. Each data collection sheet was numbered; names were not recorded to ensure anonymity. The approval of the Faculty of Health Ethics was obtained from the Biosafety and Ethics Committee of Walter Sisulu University. Written permission to conduct research at the hospital was obtained from the chief executive officer of the hospital.

## Results

For the 36-month period considered, 42 patients received ECT for catatonia.

### Demographic information

The median age was 23.5 (interquartile range [IQR] 20.1–27.0) years. Participants were predominantly male (*n* = 31; 73.81%). The sample consisted of 23 (54.76%) black patients, 18 (42.86%) mixed-race patients and one (2.38%) white patient.

### Primary diagnosis

**FIGURE 1:**
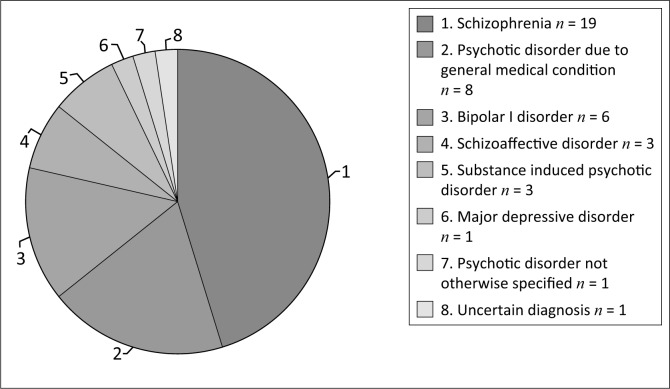
Distribution of primary psychiatric diagnoses (*n* = 42).

Thirty-four (80.95%) of the total 42 patients presented with an underlying psychotic illness. Among patients with a psychotic illness, schizophrenia was the most common diagnosis (*n* = 19; 45.24%) followed by psychotic disorder owing to a general medical condition (GMC) (*n* = 8; 19.05%) (Figure 1). Of the 8 patients diagnosed with psychotic disorder owing to a GMC, human immunodeficiency deficiency virus (HIV) was the most frequent cause, documented in 6 individuals (14.29%); epilepsy and neurosyphilis were noted as the cause in one (2.38%) patient each. Substance-induced psychotic disorder was diagnosed in 3 (7.14%) patients, with cannabis documented as the cause in 2 of these cases and methamphetamine in one of the patients. Other psychotic disorders included schizoaffective disorder (*n* = 3; 7.14%) and psychotic disorder not otherwise specified (NOS) (*n* = 1; 2.38%). Mood disorders were diagnosed in 7 (16.67%) patients. The majority of those identified with a mood disorder were diagnosed with bipolar I disorder (*n* = 6; 14.29%). Major depressive disorder was diagnosed in one (2.28%) patient. In one (2.38%) of the 42 patients in the sample, the diagnosis was documented to be uncertain.

**FIGURE 2:**
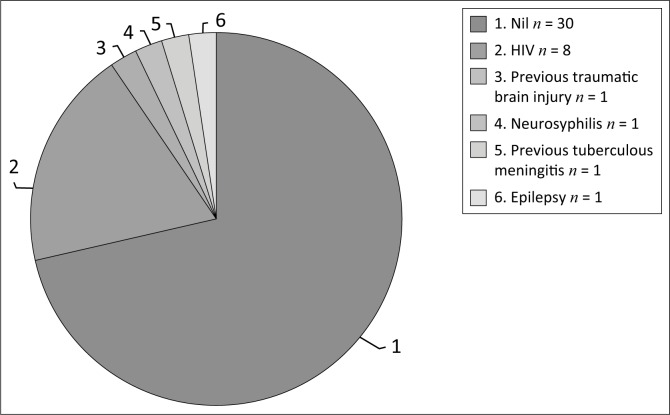
Distribution of medical comorbidities (*n* = 42).

### Medical illness

A total of 12 (28.57%) patients were diagnosed with a medical comorbidity. Eight (19.05%) patients were found to be HIV infected (Figure 2). Other medical illnesses identified included epilepsy, neurosyphilis, previous traumatic brain injury and previous tuberculous meningitis, each occurring in one (2.38%) patient; the patients with epilepsy and neurosyphilis were diagnosed as having a psychotic disorder owing to these conditions.

### Substance use

**FIGURE 3:**
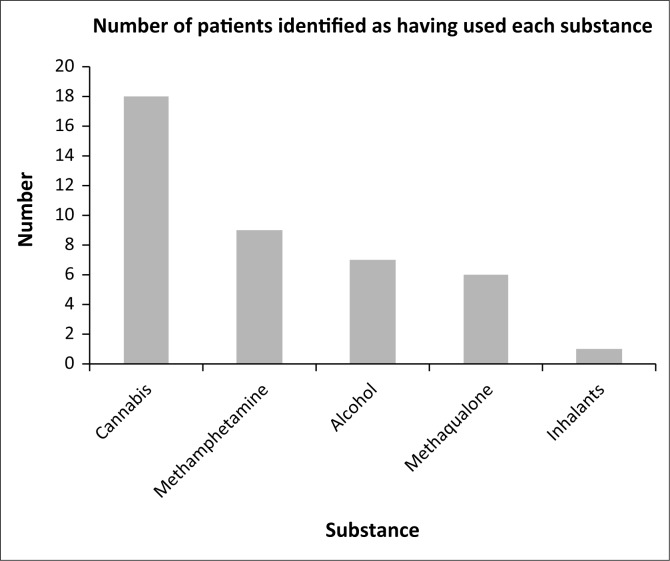
Frequency of substances used (*n* = 42).

Substance use prior to admission was identified in 20 (47.62%) patients (Figure 3). The frequency of substances used, in order of declining frequency were as follows: cannabis (*n* = 18; 42.86%), methamphetamine (*n* = 9; 21.43%), alcohol (*n* = 7; 16.67%), methaqualone (*n* = 6; 14.29%) and inhalants (*n* = 1; 2.38%). Polysubstance use, as defined by the use of 3 or more substances, was identified in 7 cases (16.67%).

### Clinical features of catatonia

**FIGURE 4:**
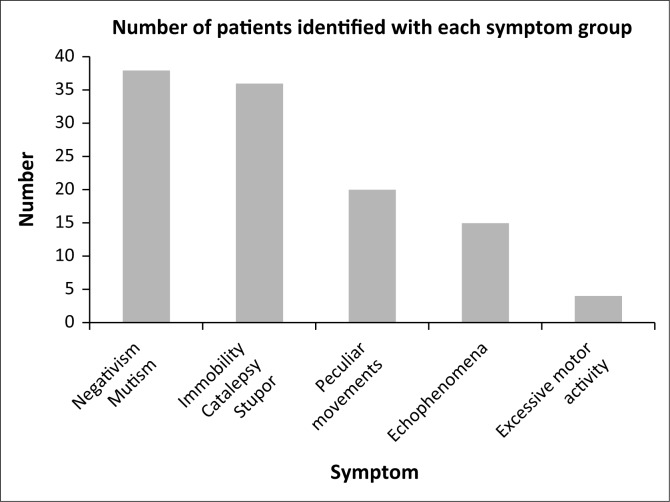
Clinical features of catatonia (*n* = 42).

The majority of patients in the sample showed features of decreased motor activity, with extreme negativism or mutism documented in 38 (90.47%) individuals and motoric immobility, including catalepsy or stupor, identified in 36 (85.71%) individuals (Figure 4). Peculiarity of voluntary movements such as posturing, stereotyped movements, mannerisms or grimacing were detected in 20 (47.61%) patients and echolalia or echopraxia in 15 (38.10%) patients. Features of excited catatonia were less common, with only 4 (9.52%) patients showing excessive motor activity. No statistically significant differences in clinical features were found in patients with different diagnoses.

### Number of electroconvulsive therapy treatments received

The median number of ECTs received was 8 (IQR 6–12). No statistically significant associations were found between demographic characteristics (age, sex or race), underlying diagnosis, or substance use, and the number of ECTs received.

### Length of hospital stay

The median length of hospital stay was 15 (IQR 10–26) weeks. Demographic characteristics (age, sex or race), underlying diagnosis and substance use were not associated with length of hospital stay.

## Discussion

### Underlying diagnosis

In this sample, psychotic disorders were more common than mood disorders: 34 (80.95%) of the total 42 patients were diagnosed with an underlying psychotic illness. Only 7 (16.67%) patients were diagnosed with mood disorders. These findings differ from those which have shown that catatonic patients frequently receive a mood disorder diagnosis,^1^ supporting the idea that catatonia may have different causes in different populations. Schizophrenia was the most common diagnosis in our sample, having been identified in 19 (45.24%) patients. These findings appear similar to those of studies that have been conducted in India. Kendurker et al. found that 59.1% of the catatonic patients in their sample were diagnosed with schizophrenia. However, this particular study investigated patients in an outpatient setting and had a small sample size.^17^ Banerjee et al. reported on 32 patients with catatonia admitted to a psychiatric hospital in India and found a higher frequency of psychotic disorders than mood disorders, with a diagnosis of schizophrenia being most frequent.^11^

### Medical morbidity

GMCs were determined to underlie the psychiatric presentation in 19.05% of the sample. All of these patients were identified with a psychotic syndrome and received the final DSM-IV-TR diagnosis of psychotic disorder owing to a GMC. No patients in the sample were identified with catatonia caused by a medical condition. It is worth mentioning that medical causes of catatonia should be considered, even when a psychiatric cause is identified, as there may be multiple causes for the catatonia.^1^ Human immunodeficiency deficiency virus was the most common illness in patients diagnosed with psychotic disorder owing to a GMC (psychotic disorder owing to a GMC was noted in 8 patients, with 6 being HIV related). Mentally ill people are vulnerable to contracting HIV.^18^ Further, HIV is known to have detrimental neuropsychiatric effects.^19^ Disturbances owing to the direct effect of HIV and/or AIDS as well as central nervous system opportunistic infections have been reported to cause catatonia.^1^ The co-existence of HIV infection and mental illness may result in marked morbidity and disease burden if not appropriately treated.^18^ Furthermore, treatments for HIV may have a number of neuropsychiatric side-effects, with reports of efavirenz causing catatonia.^20^

### Clinical features of catatonia

The majority of patients in this sample showed features of decreased motor activity with only 4 patients demonstrating excessive motor activity. No cases of malignant catatonia were identified. Negativism or mutism was identified in 90.47% of patients and features of immobility were detected in 85.71% of the sample. Peculiar voluntary movements were seen in 47.61% of patients and echophenomena in 38.10%. These findings are similar to those of Rosebush et al. who investigated 180 episodes of catatonia, at an acute facility in Canada, and found the retarded subtype to be present in the majority of cases. They also noted that close to the entire sample displayed immobility, mutism and withdrawal. Other features such as waxy flexibility and echophenomena were seen in less than 50.0% of cases.^9^ The findings of our study are also similar to those reported in an extensive review of international literature by Bhati et al., showing the most common signs of catatonia to be mutism, negativism, catalepsy, peculiar movements and echophenomena.^10^ The different underlying diagnoses in our sample were not associated with differing clinical presentations of catatonia.

### Limitations and recommendations

Identified limitations include the retrospective study design and small sample size. Rating scales were not used to support the diagnosis of catatonia or to monitor the response to ECT. It is possible that cases were missed by the treating clinicians, and it is also possible that cases were not captured on the electronic database. Although DSM-IV-TR diagnoses were recorded for the purpose of this study, clinical notes did not always contain detailed information concerning the specific criteria that the patients met for each diagnosis.

## Conclusion

In this retrospective review, we identified 42 patients who received ECT for catatonia between 01 January 2012 and 31 December 2014 at Elizabeth Donkin Hospital in Port Elizabeth, in the Eastern Cape of South Africa. The majority of patients were young adult males, aged 20–24 years. Psychotic disorders were more frequently diagnosed than mood disorders; schizophrenia was the most common diagnosis in the sample.

The findings of this study show that the diagnosis of catatonia in the Eastern Cape of South Africa is not negligible, and demonstrate the need for further local research on this topic, including the prevalence and causes. These data are required to develop clear guidelines for the management of catatonia in South Africa.
